# Molecular characterisation of the early response in pigs to experimental infection with *Actinobacillus pleuropneumoniae *using cDNA microarrays

**DOI:** 10.1186/1751-0147-49-11

**Published:** 2007-04-27

**Authors:** Jakob Hedegaard, Kerstin Skovgaard, Shila Mortensen, Peter Sørensen, Tim K Jensen, Henrik Hornshøj, Christian Bendixen, Peter MH Heegaard

**Affiliations:** 1 Department of Genetics and Biotechnology, Faculty of Agricultural Sciences, University of Aarhus, Research Centre Foulum, PO-Box 50, DK-8830 Tjele, Denmark; 2 Department of Veterinary Diagnostics and Research, National Veterinary Institute, Technical University of Denmark, Bülowsvej 27, DK-1790 Copenhagen, Denmark

## Abstract

**Background:**

The bacterium *Actinobacillus pleuropneumoniae *is responsible for porcine pleuropneumonia, a widespread, highly contagious and often fatal respiratory disease of pigs. The general porcine innate immune response after *A. pleuropneumoniae *infection is still not clarified. The objective of this study was hence to characterise the transcriptional response, measured by using cDNA microarrays, in pigs 24 hours after experimental inoculation with *A. pleuropneumoniae*.

**Methods:**

Microarray analyses were conducted to reveal genes being differentially expressed in inflamed versus non-inflamed lung tissue sampled from inoculated animals as well as in liver and tracheobronchial lymph node tissue sampled from three inoculated animals versus two non-inoculated animals. The lung samples were studied using a porcine cDNA microarray with 5375 unique PCR products while liver tissue and tracheobronchial lymph node tissue were hybridised to an expanded version of the porcine microarray with 26879 unique PCR products.

**Results:**

A total of 357 genes differed significantly in expression between infected and non-infected lung tissue, 713 genes differed in expression in liver tissue from infected versus non-infected animals and 130 genes differed in expression in tracheobronchial lymph node tissue from infected versus non-infected animals. Among these genes, several have previously been described to be part of a general host response to infections encoding immune response related proteins. In inflamed lung tissue, genes encoding immune activating proteins and other pro-inflammatory mediators of the innate immune response were found to be up-regulated. Genes encoding different acute phase reactants were found to be differentially expressed in the liver.

**Conclusion:**

The obtained results are largely in accordance with previous studies of the mammalian immune response. Furthermore, a number of differentially expressed genes have not previously been associated with infection or are presently unidentified. Determination of their specific roles during infection may lead to a better understanding of innate immunity in pigs. Although additional work including more animals is clearly needed to elucidate host response to porcine pleuropneumonia, the results presented in this study demonstrate three subsets of genes consistently expressed at different levels depending upon infection status.

## Background

Respiratory infectious diseases present a major problem in modern pig production with severe effects on both animal welfare and production economy [1]. The Gram negative bacterium *Actinobacillus pleuropneumoniae *is an inhabitant of the upper porcine respiratory tract and is the causative agent of porcine pleuropneumonia, a frequent respiratory infection which is highly infectious, often fatal and characterized by necrotizing, hemorrhagic bronchopneumonia and serofibrinous pleuritis [1]. Infection of the porcine lung with *A. pleuropneumoniae *has previously been reported to result in a local production of proinflammatory proteins or mRNA encoding the cytokines interleukin (IL) -1α, IL-1β, IL-6 and the chemokine IL-8 [2-5]. Likewise bioactive protein and/or mRNA coding for IL10, IL12p35, TNF-α˙
 MathType@MTEF@5@5@+=feaafiart1ev1aaatCvAUfKttLearuWrP9MDH5MBPbIqV92AaeXatLxBI9gBaebbnrfifHhDYfgasaacH8akY=wiFfYdH8Gipec8Eeeu0xXdbba9frFj0=OqFfea0dXdd9vqai=hGuQ8kuc9pgc9s8qqaq=dirpe0xb9q8qiLsFr0=vr0=vr0dc8meaabaqaciaacaGaaeqabaqabeGadaaakeaacuaHXoqygaqgaaaa@2E6B@ and INFα˙
 MathType@MTEF@5@5@+=feaafiart1ev1aaatCvAUfKttLearuWrP9MDH5MBPbIqV92AaeXatLxBI9gBaebbnrfifHhDYfgasaacH8akY=wiFfYdH8Gipec8Eeeu0xXdbba9frFj0=OqFfea0dXdd9vqai=hGuQ8kuc9pgc9s8qqaq=dirpe0xb9q8qiLsFr0=vr0=vr0dc8meaabaqaciaacaGaaeqabaqabeGadaaakeaacuaHXoqygaqgaaaa@2E6B@ have been shown to be up-regulated after infection with *A. pleuropneumoniae *in vivo or in vitro [2-8]. These studies have focused on a few selected genes using techniques such as quantitative real-time reverse transcriptase polymerase chain reactions (RT-PCR), northern blotting or in-situ hybridisation. The introduction of techniques for simultaneous measurements of gene expression for thousands of genes in a single analysis using microarrays allows a more comprehensive picture of the host response during infection with *A. pleuropneumoniae*. Using cDNA microarrays Moser and co-workers found 307 anonymous transcripts in blood leukocytes from pigs to be significantly affected after experimental infection with *A. pleuropneumoniae *[9].

Even though *A. pleuropneumoniae *has been extensively studied and different aspects of its pathogenesis have been described [1,2,10,11], the role of the porcine innate immune response after *A. pleuropneumoniae *infection remains poorly understood. Therefore, this response was studied further here using cDNA microarrays. Pigs were experimentally inoculated with *A. pleuropneumoniae *and microarray analyses were conducted on inflamed versus non-inflamed lung tissue from inoculated animals and on liver tissue and tracheobronchial lymph node tissue from challenged versus non-challenged pigs.

## Methods

### Animals, bacterial inoculation and samples

Six 10 – 12-week-old castrates of Danish Landrace/Yorkshire/Duroc crosses from a high health herd free from *A. pleuropneumoniae *were used in the experiment. The Danish Animal Experiments Inspectorate approved all animal procedures. Two non-inoculated animals (pigs 1 and 2) were sacrificed by means of captive bolt pistol followed by pitching and exsanguination. The animals were necropsied immediately and samples (500 mg) were taken from liver tissue and tracheobronchial lymph nodes. To investigate the effect on host responses and on the development of pathological signs of different levels of exposure to *A. pleuropneumoniae*, pigs were infected with two different doses of the same isolate. Two pigs (pigs 4 and 6) were inoculated in each nostril with 1 mL of a McFarland 0.5 suspension mixed 1:1 with Brain Heart Infusion Broth (BHI) + 0.5% NAD containing approximately 9.6 × 10^6 ^colony forming units (cfu)/mL of *A. pleuropneumoniae *serotype 5B, isolate L20 [12] and two (pigs 3 and 5) were inoculated in each nostril with 1 mL of a McFarland 0.5 suspension mixed 1:1 with BHI + 0.5% NAD containing approximately 3.8 × 10^7 ^cfu/mL of the same *A. pleuropneumoniae *isolate. The inoculated animals were sacrificed 24 hours after inoculation by means of captive bolt pistol followed by pitching and exsanguination. The animals were necropsied immediately and samples (500 mg) were taken from liver tissue, tracheobronchial lymph nodes and from both inflamed and non-inflamed lung tissue. Samples of non-inflamed lung tissue were taken as far as possible away from inflamed tissue. All samples were instantly frozen in liquid nitrogen and stored at -80°C until use. After necropsy, samples from lung, liver, tonsils and spleen were cultivated on PPLO agar (Difco, Detroit, MI, USA) to re-isolate the inoculation strain, which was serotyped using latex agglutination [13].

### Microarrays

Two-colour microarray analyses were conducted to identify genes being significantly differentially expressed in non-inflamed lung tissue relative to inflamed lung tissue sampled from the same animal, liver tissue from non-inoculated animals relative to liver tissue from inoculated animals and tracheobronchial lymph node tissue from non-inoculated animals relative to similar lymphoid tissue from inoculated animals. The microarray analyses were conducted as a common reference design in tissue-type batches. The samples of lung tissue were studied by manual hybridisation to the pig array DIAS_PIG_27K2 that contain 5375 PCR products amplified from unique cDNA clones. Samples of liver and lymph node tissues were hybridised to the pig array DIAS_PIG_55K2 (26879 PCR products) using a Discovery XT hybridisation station (Ventana Discovery Systems, Illkirch CEDEX, France). The cDNA clones used for both microarrays were selected from the cDNA libraries generated by the Sino-Danish Pig Genome Sequencing Consortium [14]. Total-RNA was purified and DNase treated using RNeasy Maxi Kit (Qiagen, Ballerup, Denmark) and aminoallyl-cDNA (aa-cDNA) was synthesized from 10 – 20 μg of total-RNA using the Superscript Indirect cDNA Labeling System (Invitrogen, Taastrup, Denmark). The obtained aa-cDNA was labelled using the ARES cDNA labelling kit (Molecular Probes/Invitrogen, Taastrup, Denmark). The reference sample was labelled with Alexa 488 and each individual sample was labelled with Alexa 594. The labelled reference samples were mixed and divided into aliquots before combining with the labelled samples. The slides were scanned and analyzed using the histogram method with default settings in a ScanArray Express HT system (version 3.0, Perkin Elmer, Hvidovre, Denmark). Statistical analysis was carried out in the R computing environment (version 2.3.0 for Windows) using the package Linear Models for Microarray Analysis (Limma, version 2.4.11, [15]) which is part of the Bioconductor project [16]. The log_2_-transformed ratios of Alexa-594 to Alexa-488 (not background corrected) were normalized within-slide using printtip-loess with default parameters. The set of normalized log-ratios were then analyzed in Limma to identify genes being significantly differentially expressed. The false discovery rate was controlled using the method of Benjamini and Hochberg [17] as implemented in Limma and a corrected P-value below 0.05 was considered significant. Spotfire DecisionSite (ver. 8.1, Spotfire, Somerville, MA, USA) was used for two-way hierarchical cluster analyses of the significantly differentially expressed genes represented by the mean log-ratios of the replicated spots (clustering method: complete linkage; similarity measure: Pearson product momentum correlation; ordering function: average value). The features of the arrays were mapped to a LocusLink identifier and an annotation package was built using the Bioconductor package AnnBuilder (version 1.9.14). A test for significantly (P < 0.05) overrepresentation of gene ontology (GO) terms among both induced and repressed genes was conducted using the GOHyperG function of the Bioconductor package GOstats (ver 1.5.5) with a threshold of minimum five genes annotated at each node. More detailed descriptions of the microarray experiments are available at the NCBIs Gene Expression Omnibus [18-20] through the GEO series accession number GSE4577.

## Results

### Necropsy findings

One pig (no. 6) died within 24 hours and by necropsy the lungs were severely affected by acute, multifocal, fibrino-necrotizing and hemorrhagic pneumonia complicated with acute diffuse fibrinous pleuritis. The tracheobronchial lymph nodes appeared enlarged and congested. No samples were taken from this animal due to autolysis. As intended, the three remaining inoculated pigs were sacrificed 24 hours after challenge and necropsied immediately. The three pigs revealed characteristic, localised, lung and pleural lesions of variable severity consistent with acute pleuropneumonia (fibrino-necrotizing pneumonia) whereas the surrounding lung tissue appeared normal. The corresponding lymph nodes of the affected lungs were enlarged and congested. The lesions in pig 4 were multifocal and up to 4 × 5 cm while the lesions in pig 3 were lobar involving most of the right diaphragmatic lobe. Pig 5 was the less affected animal with only one small (1 × 1 cm) focus of pleuropneumonia. No association was observed between inoculated dose and the severity of pathological changes and the three inoculated animals were consequently considered as one group during analyses. The inoculation strain, *A. pleuropneumoniae *serotype 5B, isolate L20, was re-isolated from lung tissue of all infected animals.

### Microarray profiling

**Figure 1:**
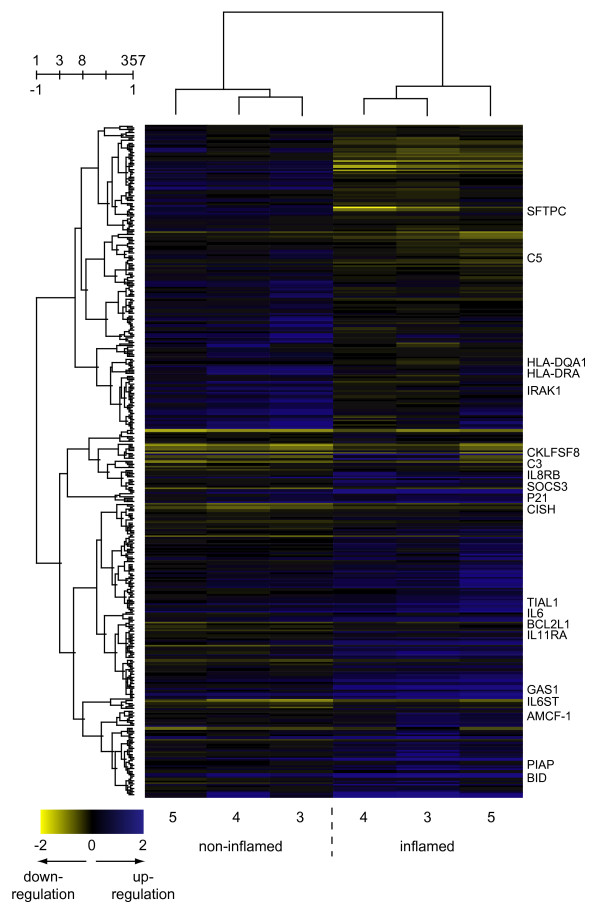
**Two-way hierarchical cluster analyses of the 357 genes affected by infection in lung tissue**. Gene expression is shown as a matrix with rows representing profiles of genes and columns representing profiles of samples. The gene dendrogram is shown to the left of the matrix and the dendrogram of the samples is shown above the matrix. Gene expression is represented by colour, with blue indicating relative up regulation and yellow indicating relative down regulation. The abbreviated gene names for selected genes are indicated to the right of the expression matrix. Numbers above the gene dendrogram represents cluster count and similarity. Text below the expression matrix represents pig number and class.

**Figure 2:**
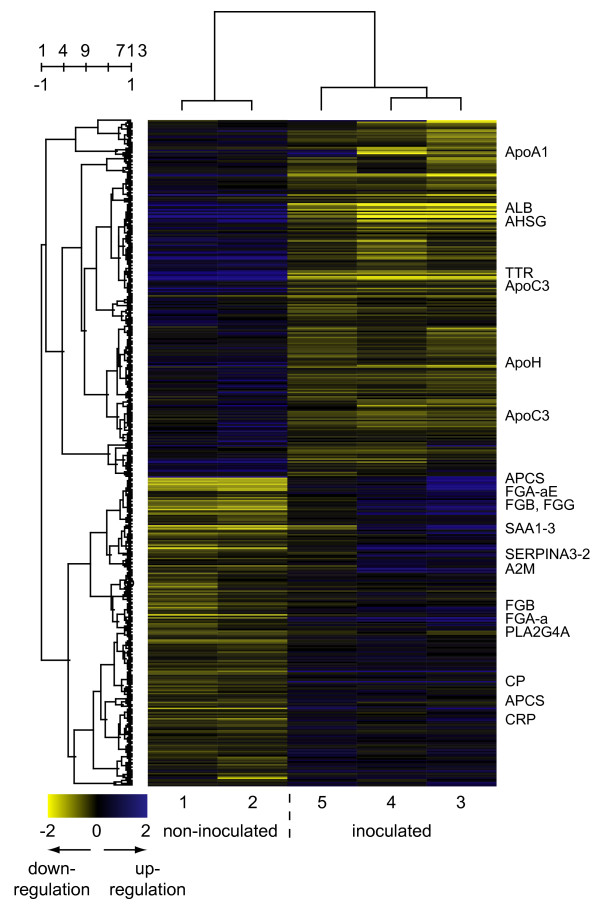
**Two-way hierarchical cluster analyses of the 713 genes affected in liver tissue by infection**. Gene expression is shown as a matrix with rows representing profiles of genes and columns representing profiles of samples. The gene dendrogram is shown to the left of the matrix and the dendrogram of the samples is shown above the matrix. Gene expression is represented by colour, with blue indicating relative up regulation and yellow indicating relative down regulation. The abbreviated gene names for selected genes are indicated to the right of the expression matrix. Numbers above the gene dendrogram represents cluster count and similarity. Text below the expression matrix represents pig number and class.

**Figure 3:**
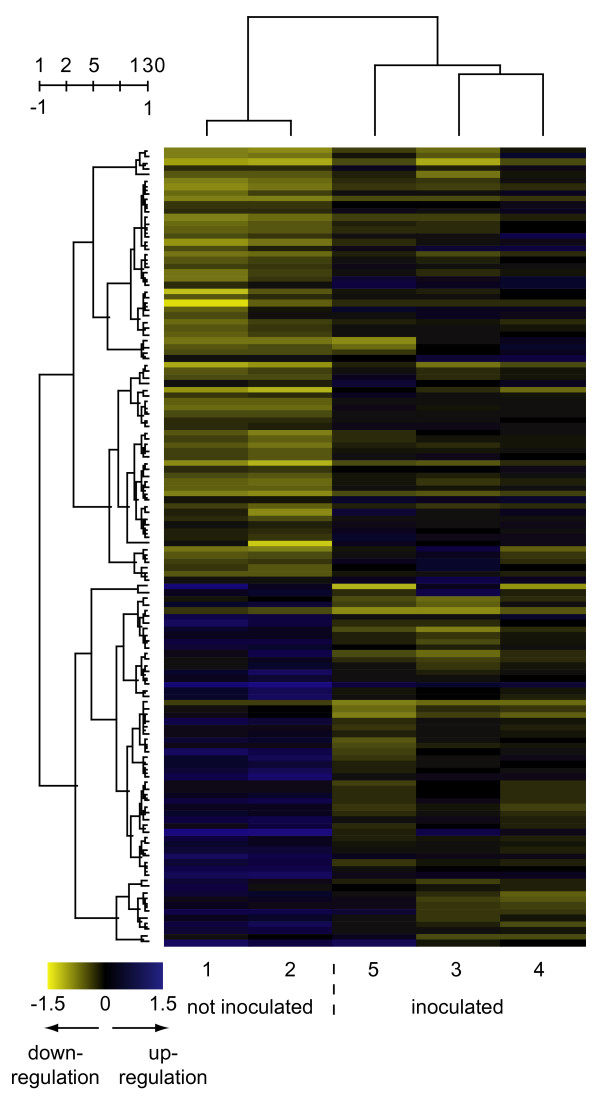
**Two-way hierarchical cluster analyses of the 130 genes affected in tracheobronchial lymph node tissue**. Gene expression is shown as a matrix with rows representing profiles of genes and columns representing profiles of samples. The gene dendrogram is shown to the left of the matrix and the dendrogram of the samples is shown above the matrix. Gene expression is represented by colour, with blue indicating relative up regulation and yellow indicating relative down regulation. Numbers above the gene dendrogram represents cluster count and similarity. Text below the expression matrix represents pig number and class.

**Figure 4:**
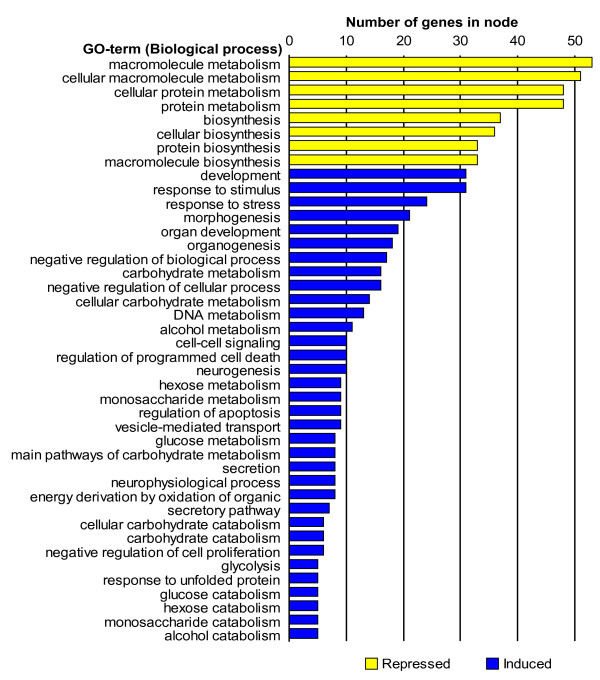
**Overrepresented GO-terms (Biological process only) among the 357 genes affected by infection in lung tissue**. The lengths of the bars represent the number of genes in each node. Repressed GO-terms are marked with yellow and induced terms by blue. Detailed descriptions of the GO terms can be found at the homepage of the Gene Ontology project [36].

**Figure 5:**
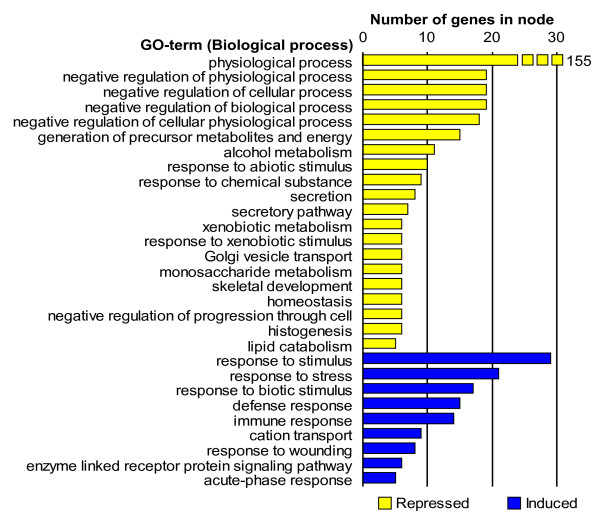
**Overrepresented GO-terms (Biological process only) among the 713 genes affected in liver tissue by infection**. The lengths of the bars represent the number of genes in each node. Repressed GO-terms are marked with yellow and induced terms by blue. Detailed descriptions of the GO terms can be found at the homepage of the Gene Ontology project [36].

Microarray analyses revealed that the experimental infection induced significant changes in the expression profiles measured in the lung, liver and tracheobronchial lymph node. A total of 357 genes (162 genes repressed and 195 genes induced) were found to be significantly differentially expressed in non-inflamed relative to inflamed lung tissue of experimentally infected pigs. The largest number of significantly differentially expressed genes was found in the liver where 713 genes were affected (382 repressed and 331 induced). In lymph node tissue, 130 genes were significantly differentially expressed with 59 genes being repressed and 71 genes being induced by the infection. It must be stressed that the lung samples were studied using a microarray with fewer genes represented compared to the microarray used for studying the liver and lymph node tissues. The lists of significantly differentially expressed genes can be found in additional file 1 "Differentially_expressed_genes". To further elucidate the effects of infection on the expression profiles in the examined tissues, two-way hierarchical clustering was applied to the mean log-ratio of the replicated spots from the significantly differentially expressed genes (Figures 1, 2, 3). As expected, the clustering revealed a clear separation of the expression profiles of the samples into two groups – one group containing profiles from inoculated animals/inflamed tissues and one group containing profiles from the non-inoculated animals/non-inflamed tissues. Expression profiles from pig 5 were seen to cluster more distantly to the profiles from pig 3 and pig 4 in all profile dendrograms of inflamed tissues/inoculated animals. Interestingly, pig 5 was the less affected animal among the inoculated animals. This indicates that the expression profiles may be associated with the severity of pathological changes. The structure of the dendrograms of non-inflamed and inflamed lung tissues (Figure 1) were found to be identical as pig 5 cluster more distantly to pig 3 and pig 4 in both. This indicates that the expression profile of non-inflamed lung tissue may be affected by the local inflammation in a distant region of the lung. Lung tissue sampled from non-inoculated pigs could hence be included in future experiments serving as an additional sample of non-inflamed tissue. The expression profiles of the genes clustered into two major groups of induces and repressed genes with several distinct sub clusters. The profiles of different cDNA fragments representing the same gene were generally observed to cluster together. The affected genes were furthermore tested for significantly overrepresentation of GO terms among both induced and repressed genes as presented below (Figures 4 and 5).

### Non-inflamed relative to inflamed lung tissue from inoculated animals

The results of the test for overrepresentation of specific GO terms among the 357 affected genes in lung tissue can be seen in Figure 4. As expected, terms related to the immune response such as "response to stimulus", "response to stress", "cell-cell signalling", "regulation of programmed cell death" and "regulation of apoptosis" were found to be overrepresented among the induced genes. Furthermore, a number of terms related to metabolism were also found to be affected.

Several of the genes observed to be induced in this study have previously been described to be induced by infection including those encoding IL-6 and IL-6 signal transducer (IL6ST), alveolar macrophage-derived chemotactic factor-I (AMCF-I) which is the porcine homologue of human IL-8 [21], IL-8 receptor beta (IL8RB), chemokine-like factor super family 8 (CKLFSF8), IL-11 receptor alpha (IL11RA), suppressor of cytokine signalling 3 (SOCS3), cytokine inducible SH2-containing protein (CISH) transcript variant 2 and complement component 3 (C3). The expression of many pro apoptotic as well as anti-apoptotic genes (encoding BCL2L1, GAS1, P21, BID, TIAL1 and PIAP) was also found to be induced in the inflamed lung tissue characterised by necrotic areas. The group of repressed genes was found to comprise those encoding members of the major histocompatibility complex (HLA-DRA, HLA-DQA1), numerous ribosomal proteins (L10 (RPL10); L11 (RPL11); L14 (RPL14); L17 (RPL17); L18 (RPL18); L19 (RPL19); L21 (RPL21); L23 (RPL23); L26 (RPL26); L27 (RPL27); L29 (RPL29); L30 (RPL30); L35 (RPL35); L37 (RPL37); S3 (RPS3); S4 X-linked (RPS4X); S7 (RPS7); S11 (RPS11); S12 (RPS12); S16 (RPS16); S19 (RPS19); S24 (RPS24); S26 (RPS26)), complement component 5 (C5), IL-1 receptor-associated kinase (IRAK1) and surfactant pulmonary-associated protein C (SFTPC). Cirera and co-workers have previously found the expression of SFTPC to be repressed in porcine lungs with necrotic areas [22].

### Liver from non-inoculated relative to inoculated animals

The test for overrepresentation of specific GO terms among the 713 genes affected by infection (Figure 5) revealed that terms related to the immune response such as "response to stimulus", "response to stress", "response to biotic stimulus", "defense response", "immune response", "response to wounding" and "acute phase response" were overrepresented among the induced genes.

As expected due to the presence of bacteria, tissue damage in the lung and host expression of IL-6, the transcripts of the following acute phase proteins were found to be accumulated in liver samples from inoculated animals: serum amyloid A1 (SAA1, transcript variant 1 and 2); serum amyloid A2 (SAA2) and A3 (SAA3); serum amyloid P component (APCS); alpha-2-macroglobolin (A2M); C-reactive protein (CRP); fibrinogen (FGA, FGB, FGG); phospholipase A2, group IVA (PLA2G4A); alpha-1-antichymotrypsin 2 (SERPINA3-2); haptoglobin (HP) and ceruloplasmin (CP). Expression of several acute phase proteins were decreased in liver samples from inoculated animals relative to non-inoculated animals including albumin (ALB), transthyretin (prealbumin, TTR), alpha-2-HS-glycoprotein (AHSG) and apo-lipoproteins (ApoC3, ApoA1, APOH). A number of these up and down regulated liver genes were validated by quantitative RT-PCR verifying these changes (data not shown, work in progress).

### Tracheobronchial lymph nodes from non-inoculated relative to inoculated animals

Even though 130 genes were found to be significantly differentially expressed in lung lymph node tissue from non-inoculated relative to inoculated animals, very few of them seem to be involved in immune response. The genes were analysed for significantly overrepresented GO terms, but the number of representative genes for each significantly overrepresented GO term was below the threshold for acceptance.

## Discussion

Transcriptional profiling using DNA microarray technology has been extensively used for studying host response to pathogenic microorganisms [23,24]. Moser and co-workers [9] studied the gene expression in porcine peripheral blood leukocytes as a response to infection by *A. pleuropneumoniae *using cDNA microarrays. A total of 18 pigs were experimentally infected with *A. pleuropneumoniae *and based on principal components analyses of seven mainly phenotypic key performance measurements, two extreme-performing animals were selected and analyzed further using cDNA microarrays. Analysis of the gene expression change from 0 to 24 hours post-challenge revealed 307 anonymous genes to be significantly affected. The results presented here are in agreement with this as numerous genes were found to be significantly differentially expressed in liver, lung and tracheobronchial lymph nodes depending on infection status.

A relative low number of genes were found to be differentially expressed in the tracheobronchial lymph nodes. This might reflect the complexity of this type of tissue compared to lung and liver tissues. Expression analysis of lymph nodes containing a variety of cell populations may lead to a dilution of the expression profile from the individual cell types. Likewise, Wurmbach and co-workers [25] found that distinguishing regulated genes from background became increasingly difficult as tissue complexity increased.

Several innate cytokines were found to be induced in inflamed areas of lung tissue from challenged animals. Significant increase of IL8 and IL6 mRNA after infection with *A. pleuropneumoniae *has previously been observed in lung lavage as well as lung tissue by northern blotting and in situ hybridisation [3,26]. SOCS3 and CISH both found to be up-regulated in the present study are members of the suppressor of cytokine signalling (SOCS) family of proteins whose members regulates protein turnover by targeting proteins for degradation [27]. The expression of the members of the SOCS family is induced by cytokines such as IL-6 and IL-10, both found to be up-regulated in this study, and function as negative feed back regulators of cytokine signalling [27,28]. The significantly increase in mRNA coding for the anti-inflammatory cytokine IL-10, found in inflamed areas of the lung, is probably due to the function of IL-10 in counteracting the host mediated tissue damage caused by proinflammatory and chemotactic cytokines [29]. The lower expression of the genes encoding ribosomal proteins could be due to a general down-regulation of ribosomal biogenesis in the necrotic areas of the lung. Previously studies have shown that 41 of 54 genes encoding ribosomal proteins were down-regulated in *Pseudomonas aeruginosa *after treatment with H_2_O_2 _inducing oxidative stress [30]. A future comparison of the expression profiles in non-inflamed lung tissue sampled from inoculated animals and lung tissue sampled from non-inoculated pigs would test this hypothesis of a lower ribosomal biogenesis in necrotic areas of the lung.

Findings of positive as well as negative regulation of acute phase proteins after infection with *A. pleuropneumoniae *seen in this study have previously been reported [31]. Serum levels of HP, CRP, and SAA increased significantly in pigs after aerosol inoculation with the same *A. pleuropneumoniae *serotype used in the present study [31]. Increased serum levels of IL-6, HP and SAA were also proven to be useful inflammatory markers for *A. pleuropneumoniae *infection in pigs [32,33]. Carpintero and co-workers found a decreased levels of ApoA1 in pig sera after 2–4 days of infection with *A. pleuropneumoniae *or *Streptococcus suis *[34]. Other affected genes known to be down-regulated during inflammation are members of the cytochrome P450 family (CYP2E1; CYP3A29) [35].

## Conclusion

The gene expression response was characterised in pigs challenged with the respiratory tract pathogen *A. pleuropneumoniae*. Although additional work including more animals is clearly needed to study the host response to this infection, the obtained results demonstrate three subsets of genes consistently expressed at different levels depending upon infection status. Two-way cluster analysis of these subsets indicated that the expression profiles of the samples may be associated with the severity of pathological changes. In inflamed lung tissue, immune activating genes and other pro-inflammatory mediators of the innate immune response were found up-regulated. In the liver of infected animals, genes that are well known to be regulated as part of the acute phase response were found to be differentially expressed. A number of genes identified in this study to be affected by infection have not previously been associated with infection or are presently unidentified. Determination of their specific roles during infection may lead to a better understanding of innate immunity in pigs.

## Competing interests

The author(s) declare that they have no competing interests.

## Authors' contributions

KS and JH contributed equally to the work and should be considered as joint first authors. JH designed and carried out the microarray analyses, conducted the statistical analysis, participated in the biological interpretation and drafted the manuscript. KS designed and carried out the experimental infections, carried out the microarray analyses, participated in the biological interpretation and in drafting the manuscript. SM carried out the experimental infections and participated in the microarray analyses. PS and HH participated in the statistical analyses. TKJ carried out the experimental infections. CB participated in drafting the manuscript. PMHH participated in the biological interpretation and in drafting the manuscript. All authors read and approved the final manuscript.

## Supplementary Material

Additional file 1 **Differentially_expressed_genes**. The file "Differentially_expressed_genes.xls" is a Microsoft Excel file and contains the worksheets "lung_uinf-inf_de-genes", "lymph_node_cont-inf_de-genes" and "liver_cont-inf_de-genes". Each worksheet contain the genes found to be significantly (fdr adjusted P-value < 0.05) differentially expressed. Click here for file
